# The Main Features and Microbiota Diversity of Fermented Camel Milk

**DOI:** 10.3390/foods13131985

**Published:** 2024-06-24

**Authors:** Zauresh Bilal, Shynar Akhmetsadykova, Almagul Baubekova, Helene Tormo, Bernard Faye, Gaukhar Konuspayeva

**Affiliations:** 1 Biotechnology Department, Al-Farabi Kazakh National University, 71 Al-Farabi Avenue, Almaty 050040, Kazakhstan; bilalzauresh@gmail.com (Z.B.); baubekova.almagul@gmail.com (A.B.); 2 LLP “Scientific and Production Enterprise Antigen”, 4, Azerbayeva Str., Almaty 040905, Kazakhstan; shynar.akhmetsadykova@gmail.com; 3 LLP “Kazakh Research Institute for Livestock and Fodder Production”, Horse and Camel Breeding Department, 51, Zhandosov Str., Almaty 50035, Kazakhstan; 4 Département Sciences de l’Agroalimentaire et de la Nutrition, Ecole D’ingénieurs de Purpan, INPT, 75, voie du TOEC, BP 57611, CEDEX 3, 31076 Toulouse, France; helene.tormo@purpan.fr; 5 Center of International Cooperation on Agriculture Research for Development–CIRAD, UMR SELMET, Campus International de Baillarguet, CEDEX 5, 34398 Montpellier, France; bjfaye50@gmail.com

**Keywords:** *shubat*, Kazakhstan, camel milk, fermentation, microbiota

## Abstract

Fermented camel milk, named *shubat* in Central Asia, is historically and culturally important because it is mainly consumed by Kazakh people who live not only in Kazakhstan but also in close neighboring countries. However, despite its cultural and dietetic significance for this local population, research on its composition and processing technology and the richness of its microflora is relatively scarce. The present review of this product, which is an important beverage in the Kazakh culture, provides up-to-date information regarding its main components and their variability according to different factors, surveys recent changes in the processing technologies for making it using modern techniques, and explores the biodiversity of its microflora. It was reported that the protein, vitamin C, and calcium contents in shubat vary between 1.19 and 5.63%, 28 and 417 mg L^−1^, and 1.03 and 1.88 g L^−1^. The lactose content totally disappears. *Shubat* contains a complex microbial consortium that contributes to its strong reputation for health benefits, but a scientific demonstration of these claims has only been partially achieved.

## 1. Introduction

The consumption of fermented dairy products was historically a common feature, especially in Central Asia [1], because it was one of the best solutions to prolong the shelf life of milk from species such as horses or camels, for which the traditional processing of milk into cheese was problematic [2,3]. Fermented camel milk, named *shubat* in Kazakhstan, can be regarded as an “identity product” by the Kazakh population worldwide [4]. *Shubat* is a slightly acidic liquid dairy product that resembles a drinkable yogurt (only in a liquid form). It is consumed throughout the day, mainly after meals. However, despite its cultural and dietetic importance for this local population, research on its composition and processing technology and the richness of its microflora is relatively scarce. The present review aims to provide up-to-date information about these topics, including (i) the place of *shubat* in the culture of the Kazakh population; (ii) its main components and their variability; (iii) the current changes in the processing technologies for making *shubat* using modern techniques; and (iv) the biodiversity of its microbiota. 

## 2. Geographical and Historical Aspects of *Shubat* Distribution

*Shubat* is historically and culturally important in Central Asia because it is mainly consumed by Kazakh people who live not only in Kazakhstan but also in close neighboring countries such as Russia, China, Kyrgyzstan, Uzbekistan, Turkmenistan, Azerbaijan, and Iran, as well as more distant countries such as Turkey, Mongolia, Tajikistan, and Afghanistan. The Kazakh population normally uses spontaneous fermentation of camel milk in spring after a long selection process to obtain the traditional taste of *shubat*. Thus, it is possible to consider that the *shubat* season starts at the end of spring and the beginning of summer. Usually, *shubat* production is continued after snowfall [1]. 

Historically, *shubat* was produced in all areas practicing camel breeding. Before the Soviet era, camel breeding was observed in the whole territory of Kazakhstan. Later, during the 20th century, camel farms were mainly implemented in the south and southwestern regions of Kazakhstan, where there is a more arid climate. *Shubat* consumption accompanied all the main steps of Kazakh people’s lives from an ethnographical point of view. This tradition was only preserved in arid zones during the Soviet era. After independence in 1991, the society returned to tradition, widely consuming *shubat* at all traditional festivals and events, such as 40 days after birth, circumcision, starting school, birthdays, the traditional funeral dinner, 40 days after a funeral, and the first anniversary of a death [5]. The consumption of *shubat* at traditional festivals and events is considered a Muslim tradition because the consumers believe that this beverage does not contain alcohol. However, regarding the amount of alcohol in *shubat,* there are still few data available, even if, theoretically, it is supposed that a small amount of alcohol could be present. For this reason, *shubat* is widely accepted, even today, in all new independent states (where Kazakh people are living).

## 3. Local and International Regulations Regarding *Shubat*

During the Soviet era, many standards and normative documents were established by each Soviet republic for camel milk and its products. In the Kazakh SSR (actual Kazakhstan) in the 1980s, a state standard for the camel milk used (as a raw material) to produce *shubat* was established, and a separate state standard for *shubat* itself was created [6]. In the first version of the standard regarding the camel milk used to prepare shubat, it was mentioned that it was possible to have different versions of *shubat*: “soft”, “medium”, and “strong”. This differentiation was mainly based on the acidity level. It must be noted here that the Kazakh population prefers the fermented milk to be strongly acidic. In many cases, the acidification measured using the Thurner method reaches around 165 degrees, corresponding to “medium”, while the acidity of *shubat* usually varies between 106 and 215 degrees [7].

After the independence of Kazakhstan, these standards were updated (between 1991 and 2010). However, since the creation of the Customs Union between Kazakhstan, Russia, Belorussia, and others in 2011, all normative documents were progressively updated to reflect Customs Union norms. Regarding camel milk and *shubat* standards, Kazakhstan was obliged “to come back” to old Soviet standards edited in 1986 (second edition) [8]. 

The Soviet standards for *shubat* were the first in the world. Indeed, even in the *Codex Alimentarius* of the FAO/WHO, camel milk was only mentioned in 2006. Since that time, no technical documents have been proposed at the international level. Currently, camel milk and all its products need to adhere to food safety norms without specification of the type of milk or product.

## 4. Composition of *Shubat*

The composition of *shubat* strongly depends on the camel milk’s composition and the fermentation processes that were used to produce it. In the literature, there is a total absence of global composition, and for that reason, no mention of *shubat* is made. Globally, the mean physico-chemical parameters of *shubat* from the southeastern, southern, southwestern, and western regions of Kazakhstan, taken during a whole-year survey, showed wide seasonal, regional, and specific variability, as reported by Konuspayeva [7] and Narmuratova [9]. Notably, they observed significant effects of region and season on proteins, vitamin C, and the calcium content, probably related to the variability in the camels’ diets (region/season) and lactation stages (season). Indeed, due to the seasonal reproductive cycle of camels, winter *shubat* is made with milk at the beginning or end of lactation, while summer *shubat* is made with milk at the peak of lactation. The mean milk composition varies strongly according to region and season; the impacts on *shubat* composition are obvious [7]. On average, the protein, vitamin C, and calcium contents vary between 1.19 and 5.63%, 28 and 417 mg L^−1^, and 1.03 and 1.88 g L^−1^ (Table 1). Lactose (determined by the NF V 04-213 method) totally disappears from all *shubat* samples [7,9].

It is important to emphasize the very high level of vitamin C in raw and fermented camel milk in Kazakhstan [10], especially in the summer *shubat* from the western part of the country [11]. It may be one of the main explanations for the health claim reported by local consumers. The fatty acid composition and its regional and seasonal variation were also determined, showing a low proportion of short-chain fatty acids and high contents of myristic (C14), palmitic (C16), stearic (C18), and oleic acid (C18:1), while linoleic acid (C18:2) was only present in one sample [9].

## 5. *Shubat* Process from Traditional to Modern Technology

In southern Kazakhstan, *shubat* is called *qymyran*. It is prepared with boiled (pasteurized) camel milk. In some other regions, the *shubat* processed in summertime is also called *qymyran* and is characterized by a more liquid texture and a low fat content [1]. The traditional method of preparing *shubat* is quite simple: it is made by adding a small quantity of previously soured milk to fresh unprocessed (raw) camel milk. The fermentation process is performed for 1–2 days in a specialized container made of skin or wood [12] and may be the main source of starters for dairy products [13]. This kind of *shubat* preparation on camel farms does not change globally. Fresh camel milk collected throughout the whole day is mixed with previously soured milk and stored at room temperature for 1–2 days. This traditional technology is used at small-scale manufacturing sites organized by individual farmers or cooperatives, which produce the majority of *shubat* in Kazakhstan [4].

However, with the modernization of agriculture, a larger variety of camel dairy products have appeared (other than *shubat*), the quality of the products has improved (such as pasteurized camel milk), and new technologies and rational methods for processing raw materials have been introduced (such as powdered camel milk, cheeses, and ice cream). Recently, most of the traditional fermented products have undergone serious modernization in their processes in response to agricultural industrialization. Therefore, the modernization of *shubat* technology began relatively recently due to the increasing demand for a more standardized product [14]. 

However, the “modern technology” involved in *shubat* preparation varies depending on the manufacturer and region. The most common process consists of the following operations: Fresh camel milk is filtered, then pasteurized at a temperature that varies from 63 °C to 90 °C for a duration of 5 min to 5 h. Some mini dairy plants (private) exceed this heating time without any technical or scientific augmentations. Then, after cooling to 30–35 °C or room temperature, the milk is poured into an oak (or plastic or inox) barrel and finally sowed with the starter culture, which is added at a ratio of one part of starter (mother) culture to three to four parts of pasteurized camel milk (but the use of raw camel milk still exists). Then, for 20–30 min, the mixture is thoroughly kneaded with a whorl and left to ferment for 3–4 h at 20–25 °C. During this time, the acidity increases and reaches 60–70 °T. The *shubat* must be kneaded frequently, breaking up the casein particles. Since camels are milked three times a day (in summertime), fresh camel milk is added to a barrel with a fermenting mixture, “rejuvenating” the drink each time. Fermentation occurs at 20–25 °C for 10–20 h up to approximately 125 °T. After that, the drink is packaged in bottles, capped, and placed in a refrigerator for 10–12 h for ripening. The shelf life of the final product at 5–10 °C is 5–6 days. Depending on the duration of lactic acid fermentation, *shubat* is divided into three categories: weak—maturation within a day (60–80 °T), medium—maturation within two days (80–105 °T), and strong—maturation with three days (106–125 °T). These categories of *shubat* accordingly contain 0.5% to 1.2% alcohol [15] (State Standard of the Republic of Kazakhstan ST RK 166-97 “Camel milk for processing into shubat”, valid until 01/2017).

*Shubat* preparation technology may vary depending on the camel dairy farms, regions, and seasons. In summer, the fermentation of camel milk into *shubat* is faster compared to spring, when the starter’s composition is not yet well stabilized. The preparation technology and the seasonal variability in camel milk composition could explain the different tastes, smells, and textures observed in the end products. Thus, in some regions, *shubat* appears viscous and dense, while in other regions, it can be a liquid with gas and foam [1].

Zhusipova [16] developed and patented a freeze-dried tableted *shubat* technology. These tablets can be crunched like sweets. This kind of product can be stored for more than 1 year and transported over long distances.

Another research team patented a sachet granule technology for freeze-dried shubat. This method allows one to obtain high-quality *shubat* with mass fractions of 86.0% moisture, 4.75% protein, and 4.65% fat; maximum preservation of vitamins A (0.46 mg 100 g^−1^), E (0.15 mg 100 g^−1^), and C (7.9 mg 100 g^−1^); and an acidity of 95 °T. According to the authors, this technology allows one to retain the organoleptic properties of *shubat* after cryo-sublimation. Sachet granules can be used as medicinal food additives and are available for the microbiological and pharmacological industries [17].

Combined fermented milk drinks based on camel milk are being developed to expand the variety of food products that consider national traditions and the taste of the population. Various additives of vegetable origin (pumpkins, carrots, and beets) can be added to enrich the final products with biologically active substances, vitamins, and carbohydrates, providing a specific taste [18,19]. *Shubat* with fruit juices (sea buckthorn or peach) has also been proposed [20], along with herbal supplements in the form of pumpkin seeds, parsley, dill, and basil [21] or extracts of medicinal plants such as peppermint (*Mentha piperita* L.) [22].

## 6. Microbiology of *Shubat*

*Shubat’s* natural microbial community includes lactic acid bacteria (LAB), yeasts, and sometimes molds [23,24,25,26]. As a result, like other traditional fermented products, there are two types of fermentation: lactic and alcoholic [27]. The interaction between these microbial populations (LAB, yeasts, and molds) is probably the reason for the distinct sensory characteristics and physical properties of *shubat*. In addition, the metabolic processes of these microorganisms, particularly lactic acid fermentation and possibly partly alcoholic fermentation, play a role in preserving *shubat* and may produce bioactive metabolites that provide health advantages [27].

### 6.1. Bacterial Community 

The abundance of presumed LAB (counted on culture media) is around 107 to 108 CFU mL^−1^, and the abundance of yeasts is around 104 to 107 CFU mL^−1^ [26,28]. According to the abundance of yeast and the mixed fermentation of LAB, ethanol is detected in most samples (0.6% to 1.2%) (State Standard of the Republic of Kazakhstan ST RK 166-97 “Camel milk for processing into *shubat*”, valid until 01/2017). 

However, regarding the major LAB communities, some differences arise depending on the identification method: culture-dependent or -independent approaches, e.g., metagenetic or metagenomic. To obtain a complete picture reflecting the core microflora of *shubat* and the role of individual culture in the ripening of the product, special attention should be paid to the study of its microflora using different identification methods that complement each other. For a more complete overview of bacterial communities, 16S rRNA gene sequencing, in some cases using a metagenomic approach, is one of the best tools currently used. For example, concerning the microbial diversity of *shubat* investigated using amplified full-length 16S rRNA genes, Yu et al. [29] suggested that the species richness (Chao index) and the diversity in *shubat* from Inner Mongolia were high (333 ± 138 and 2.96 ± 0.55, respectively). At the same time, with amplified V3–V4 16S rRNA genes, the Chao index and microbial diversity of *shubat* in Kazakhstan were much lower (51 ± 3 and 1.41 ± 0.41) [30], indicating that these values may vary depending on the test method and the hypervariable regions of the 16S rRNA gene used. Elsewhere, identification of strains isolated from culture media has the disadvantage of promoting specific microorganisms rather than others, depending on the culture condition. However, this method is necessary to validate the viability and growth of bacteria, identify strains, and study their properties.

Considering both approaches, the main bacteria identified in *shubat* belong to the genera *Lactobacillus*, *Lactococcus*, *Leuconostoc*, and *Enterococcus*, which were detected using the culture-dependent method (Table 2). For the samples considered in different studies (Table 2), 39 bacterial species were detected in *shubat*, predominantly from the families *Lactobacillaceae*, *Enterococcaceae*, and *Streptococcaceae*, with isolated species from the families *Acetobacteraceae*, *Hafniaceae*, *Moraxellaceae*, and *Burkholderiaceae*.

**Table 2:** Microbial diversity of *shubat* in 20 studies.

	[31]	[26]	[28]	[32]	[23]	[24]	[33]	[25]	[34]	[30]	[29]	[35]	[36]	[37]	[38]	[39]	[40]	[18]	[41]	[42]
Bacteria																				
Enterococcus faecium *		+			+	+		+	+								+			
Enterococcus faecalis *		+			+	+		+												
Lacticaseibacillus casei *	+		+	+	+		+		+						+	+				
Lactobacillus helveticus *		+	+					+		+	+			+				+		
Lactiplantibacillus plantarum *			+						+				+		+					
*Lactobacillus delbrueckii* subsp. Bulgaricus *	+									+	+				+					+
Lactococcus lactis *				+		+						+			+		+			+
Latilactobacillus sakei *		+			+			+												
*Leuconostoc mesenteroides* subsp. Mesenteroides *					+	+				+										
Leuconostoc lactis *		+						+												
Levilactobacillus brevis *		+						+												
Limosilactobacillus fermentum *				+										+	+					
Lacticaseibacillus paracasei *							+		+											
*Weissella hellenica*		+						+												
*Pediococcus acidilactici*	+								+											
*Lentilactobacillus kefiri*										+			+							
*Acetobacter pasteurianus*						+				+										
*Enterococcus durans*					+	+														
*Enterococcus hirae*					+															
*Enterococcus lactis*									+											
*Lactobacillus kefiranofaciens*											+		+							
*Bifidobacterium mongoliense*													+							
*Lentilactobacillus buchneri*					+															
*Lentilactobacillus curieae*									+											
*Limosilactobacillus pontis*																		+		
*Limosilactobacillus reuteri*																				
*Moraxella osloensis*										+										
*Pediococcus pentosaceus*									+											
*Secundilactobacillus oryzae*									+											
*Streptococcus salivarius*											+									
*Streptococcus thermophilus*										+										
*Weissella confusa*									+											
*Yeast*																				
Kazachstania unispora *		+			+			+												
Kluyveromyces marxianus *		+		+	+			+												
*Candida ethanolica*		+							+											
*Brettanomyces bruxellensis*					+															
*Galactomyces geotrichum*					+															
*Naumovozyma castellii*						+														
*Saccharomyces cerevisiae*					+															
*Saccharomyces lactis*																	+			
*Candida kefyr*								+												
*Brettanomyces anomalus*																			+	

* indicates most abundant microorganisms in *shubat*.

The identification of camel microbiota using the culture-dependent method shows that *shubat* contains LAB belonging mainly to the genera *Enterococcus*; *Lactobacillus*; *Leuconostoc*; and, less frequently, *Pediococcus* (Table 2, list of main authors). In studies using metagenetic or metagenomic methods, *Lactobacillus* was found most frequently and was the most abundant genus in the 11 *shubat* samples studied by the authors of [29,30,35,36]. For these studies, *Lactobacillus helveticus* (up to 75%), *L. delbrueckii*, *L. kefiranofaciens*, and *Lentilactobacillus kefiri* were the most abundant, with high variability between samples and studies. *S. thermophilus* was often detected with *L. delbrueckii*, suggesting the use of yoghurt as a starter in some samples. *Lactoccoccus lactis*, *Leuconostoc mesenteroides*, and *Streptococcus salivarius* were found in some samples, with lower abundance than *Lactobacillus*. *Enterobacteriaceae* was found in some samples (*Hafnia alvei* and *E. xiangfangensis*). Interestingly, Elcheninov et al. [36], using shotgun metagenomic analysis, found *Bifidobacteriaceae* (*Bifidobacterium mongoliense*) in one *shubat* sample. Surprisingly, *Enterococcus* was not found, although it was often isolated using the culture-dependent method in *shubat* and many types of naturally fermented milk [23,26,31]. A possible reason for this is that *Enterococcus* grew well in culture media compared to other species. If we refer to Yu et al. [29] and Zhadyra et al. [30], the bacterial composition of *shubat* differed slightly from those of other types of fermented milk (notable overabundance of *L. helveticus* in *shubat*), but 33 species were common with *ayran* (among the 50 species in *shubat*) and 360 OTUs were common with *qymyz* (fermented mare milk). Moreover, Yu et al. [29] identified 10 OTUs in samples of all eight types of spontaneously fermented dairy products, including *S. salivarius*, *L. helveticus*, *L. delbrueckii*, *E. xiangfangensis*, and *Acinetobacter baumannii*. Therefore, it was inferred that these five species represented the primary bacteria in the spontaneously fermented dairy products. 

Various LAB species exhibit distinct qualities that, during fermentation, assist in breaking substrates into metabolites, contributing to the unique taste and aroma of *shubat* [30,43]. Both types of bacteria, homofermentative and heterofermentative, are found in *shubat* (Table 3). *Leuconostoc mesenteroides*, *Lentilactobacillus kefiri*, *Lacticaseibacillus casei*, *Lactilactobacillus sakei*, and *Lactobacillus casei* subsp. *casei* produce D- and L-lactate, carbon dioxide, ethanol, and acetate [44,45,46].

The effects of D- and L-lactate on human organisms differ completely. L-lactate is metabolized by L-lactate dehydrogenase (LDH), which is naturally present in the human intestine, whereas the breakdown of D-lactate occurs five times slower, and its accumulation can lead to D-lactic acidosis [47].

**Table 3:** Fermentation characteristics of major microorganisms in *shubat*.

Microflora	Type of Fermentation	Substrate	Enantiomeric Form of Lactic Acid	Primary Metabolites	References
*Acetobacter pasteurianus*	Acetic acid	Ethanol + lactate		Acetate + acetoin methanol	[48,49]
*Enterococcus durans*	Lactic acid (homofermentation)	Lactose, glucose, and others	L(+)	Lactate, acetoin	[50,51]
*Enterococcus faecalis*	Lactic acid (homofermentation)	Lactose, glucose, and others	L(+)	Lactate, acetoin	[52,53]
*Enterococcus faecium*	Lactic acid (homofermentation)	Lactose, glucose, and others	L(+)	Lactate, acetoin	[54]
*Lactobacillus delbrueckii* subsp. *bulgaricus*	Lactic acid (homofermentation)	Lactose, glucose, galactose, and others	L(+)	Lactate	[55]
*Lacticaseibacillus paracasei* and *Lacticaseibacillus casei*	Lactic acid (homofermentation in general, but some strains could be facultatively heterofermentative)	Glucose, fructose, mannose, galactose, lactose, cellobiose, and trehalose	L(+) and sometimes D(−)	Lactate (major compounds) and for some strains ethanol and/or acetate, CO_2_/diacetyl, acetoin	[45,56,57]
*Lactobacillus helveticus*	Lactic acid (homofermentation)	Glucose, lactose, mannose, and trehalose	L(+), D(−)	Lactates	[58,59]
*Lactococcus lactis*	Lactic acid (homofermentation)	Galactose, glucose, fructose, lactose, and others	L(+)	Lactate/folate gamma-aminobutyric acid	[59,60]
*Latilactobacillus curvatus*	Lactic acid (homofermentation)	Galactose, glucose, and others	L(+), D(−)	Lactates	[61,62]
*Latilactobacillus sakei*	Lactic acid (facultative heterofermentation)	Glucose or ribose	L(+)	Lactate, acetate, aroma compounds (diacetyl and acetoin), ethanol	[59,62,63,64]
*Lentilactobacillus kefiri*	Lactic acid (facultative heterofermentation)	Lactose, glucose, galactose, and others	D(−)	Lactate, ethanol, acetate, CO_2_	[46]
*Leuconostoc lactis*	Lactic acid (facultative heterofermentation)	Lactose, maltose, D-glucose	D(−)	Lactate, ethanol and/or acetate, CO_2_, acetoin	[65,66,67]
*Leuconostoc mesenteroides*	Lactic acid (facultative heterofermentation)	D-fructose, D-glucose, D-mannitol, D-mannose, L-arabinose, lactose, maltose, sucrose	D(−)	Lactate/dextran, class C polysaccharides	[44,65,68]
*Levilactobacillus brevis*	Lactic acid (obligatory heterofermentation)	Hexoses, pentoses, glycerol	D(−)	Lactate, acetate, ethanol, CO_2_, 1,3-propanediol	[69,70]
*Streptococcus thermophilus*	Lactic acid (homofermentation)	Lactose, sucrose, galactose	L(+)	2 lactate +, folate, acetoin	[71]
*Candida ethanolica*	Alcohol	D-glucose; ethanol; glycerin (slowly); and lactic, succinic, citric (weakly), and gluconic acids		Glycerol, acetic acid, ethanol	[72]
*Dekkera anomala*	Alcohol	D-glucose, D-galactose, sucrose, maltose, trehalose, c~-methyl-D-glucoside, + cellobiose, lactose No lactate assimilation		Glycerol, acetic acid, ethanol, phenolic compounds	[73] CIRM Levures (www.bio-aware.com, accessed on 13 May 2024)
*Dekkera bruxellensis*	Alcohol	Fermentation of D glucose and D galactose Carbon assimilation: Lactose-galactose-glucose + lactate-, Citrate-		Glycerol, acetic acid, ethanol, volatile phenols 4-ethylphenol and 4-ethylguaiacol	[74] CIRM Levures (www.bio-aware.com, accessed on 13 May 2024)
*Galactomyces geotrichum*		Fermentation (yeast data base): D Glucose +/− Lactose – D galactose – Carbon assimilation: Glucose, galactose, lactate, citrate	L(+)	Lactic acid, phenylacetaldehyde, phenylacetic, phenyllactic acid	[75,76] Confirmed by theyeasts.org, accessed on 13 May 2024
*Kazachstania unispora*	Alcohol fermentation Non-lactose-fermenting yeast	D-Galactose, D-Glucose	-	Succinic acid (umami taste), low ethanol production	[77,78,79,80]
*Kluyveromyces marxianus*	Alcohol fermentation Lactose-fermenting yeast	Glucose (+), galactose (+), lactose (+; −), sucrose (+)	-	Ethanol, 2-phenylethanol	[81,82,83,84] https://wi.knaw.nl/, accessed on 13 May 2024
*Saccharomyces cerevisiae*	Non-lactose-fermenting yeast, glycolytic pathway, alcoholic fermentation	D-Galactose, maltose, sucrose, raffinose	-	Lactate, ethanol, and/or acetate, CO_2_ Production of ethanol and other byproducts	[78,85,86]

Ultimately, these results show that the microbiota of *shubat* is an intricate ecological system mostly controlled by LAB, specifically *Lactobacillus* species, which are responsible for fermentation and may provide supplementary capabilities. Further investigation employing omics methods could provide a more holistic understanding of this distinctive microbial population and its prospective applications.

### 6.2. Yeast Community

As shown in Table 3, the identified yeasts belong to both lactose-fermenting and non-lactose-fermenting species, as well as species producing alcoholic fermentation. There is a significant shortage of research on the yeast characteristics of *shubat*. Nevertheless, studies revealed the presence of the species *Kazakhstania unispora*, *Kluyveromyces marxianus* (most often cited), *Candida kefir*, *Candida ethanolica*, *Dekkera anomalus*, *Dekkera bruxellensis*, *Naumovozyma castellii*, *Saccharomyces lactis*, *Saccharomyces cerevisiae*, and *Galactomyces geotrichum* (Table 2). 

*K. unispora*, *Kl. marxianus*, *C. kefir*, *C. ethanolica*, *S. lactis*, and *S. cerevisiae* are species of the autochthonous microbial population of traditional dairy products, particularly *kefir*, the traditional fermented milk that has received the most scholarly attention [87,88]. Apart from *K. marxianus*, the other species are non-lactose-fermenting in milk (Table 2). The abundance of these species in fermented milks and *shubat* suggests a symbiotic relationship between lactose-fermenting yeasts and LAB. Liu et al. [89] demonstrated that *C. kefyr*, *K. marxianus*, and *Galactomyces geotrichum* exhibit stability-enhancing effects on *Lactobacillus* species (*L. delbrueckii* and *L. rhamnosus*). Yeasts could have a significant impact on aroma compounds in fermented milk. The aroma of *qymyz*, for example, could be modulated by *K. unispora* [77]. Recent studies have highlighted the sensorial advantage of the mixed fermentation of LAB (*L. brevis*, *L. delbrueckii*, and *S. thermophilus*) and *K. marxianus* (with or without the addition of *S. cerevisiae*) for fermented milk. The metabolic pathways of flavor-related substances can be changed in a positive way [90,91]. This affects the synthesis of aminopeptidases, which reduces bitterness, as it does in cheese [76,92]. To our knowledge, the importance of *G. geotrichum* in traditional fermented milk has not been highlighted [93].

Recent studies have underlined the role of yeasts as promising probiotics. For example, *K. marxianus* has the capacity to modify cell immunity, adhesion, and human gut microbiota and has antioxidative, anti-inflammatory, and hypocholesterolemic properties [78,94].

The diversity of the yeasts detected in *shubat* should be considered. The implications for sensorial features, interactions between lactic acid bacteria, probiotic properties, and shelf life are interesting subjects for future analysis.

## 7. Probiotic Properties of *Shubat*

Some of the initial data in the scientific literature reporting the therapeutic properties of *shubat* appeared in the second half of the 20th century [95,96,97]. Its effectiveness has been noted in the treatment of chronic diseases of the digestive system such as gastritis, peptic ulcers, liver diseases, and large and small intestine disorders. When they regularly consumed *shubat*, patients felt better, pain disappeared, appetite increased, the secretory function of the stomach normalized, and the motor evacuation function of the small intestine normalized with a decrease in secretion [31].

There have been recent studies on the therapeutic effect of *shubat* produced by Kazakhs living in Xinjiang. It was found that *shubat* had significant anti-inflammatory effects. This anti-inflammatory mechanism was associated with inhibition of the infiltration of inflammatory factors, a reduction in CRP, elimination of free radicals, and inhibition of lipid peroxidation in animal experiments [98].

Scientists noticed that ethnic Kazakhs, for whom *shubat* was a constant component of their diets, suffered from type 2 diabetes to a lesser extent [99]. It was suggested that this might be due to the daily consumption of fermented foods, including *shubat*. A study on the therapeutic effects of probiotic fermented camel milk in rat models of type 2 diabetes mellitus, induced by administration of a high-glucose and high-fat diet and a low dose of streptozotocin, revealed marked decreases in fasting blood glucose and HbA1c and increases in C-peptide and GLP-1 levels. In addition, there were improvements in renal function and lipid metabolism, as well as an improvement in pancreatic β-cell function [99]. The authors have not ruled out the possibility that this was due to the action of various probiotics that promote the release of GLP-1 and improve β-cell function. The effect of probiotics was more clearly demonstrated in experiments in mouse models, where it was shown that 14 probiotic strains isolated from fermented camel milk had positive effects on blood glucose and lipids, delaying the development of T2DM and stimulating the secretion of GLP-1 [100]. In addition, 4 of these 14 probiotic strains of microorganisms were found to alleviate cyclophosphamide-induced immunodeficiency in mice and exert antitumor effects by regulating the CD4+/CD8+ cell ratio and increasing the secretion of Th1-type cytokines [101]. In addition to probiotics, bioactive peptides isolated from *shubat* from Xinjiang were found to have anti-inflammatory and immunomodulatory functions, as well as stimulating effects on mouse splenic lymphocyte proliferation and the expression of IFN-γ in lymphocytes [102].

Obviously, research on *shubat* is limited in general, and research on its therapeutic effects is even less common. Hence, despite the wide use of *shubat* in Kazakhstan and by Kazakhs living in Western China, this product is understudied in terms of its microbiology, nutritive value, and “therapeutic” effect.

Like many traditional fermented beverages, *shubat* contains a complex microbial consortium and has a strong reputation for health benefits, but scientific demonstrations of these claims have only been partially achieved. *Shubat* is produced by the ancient practice of “backslopping”. Thus, there is continuous selection and adaptation of microbes in the human-created fermentation environment. The microbiota of these products may represent genetically distinct and isolated clades in the LAB and yeast family trees compared to the same species of commercial strains.

## 8. Conclusions

*Shubat* is a typical product from Kazakhstan and can be regarded as an ethnic food with high dietetic value and potential health effects. The modernization of its processing technology and attempts to obtain a *shubat* more adapted to modern life in urban areas (new tastes, new packaging, proved health claims, and potential geographical origin identification) require a better understanding of the richness of its microbiota and its variability in terms of location and time of year. These fermented dairy products represent complex microbial ecosystems, and further research should focus on (i) the link between the technologies used to prepare *shubat* and the compositions of the final products, including microfloral biodiversity, aromas, and tastes; (ii) the investigation of microfloral biodiversity and its impact on the health of human consumers, including clinical trials; (iii) the possibility of implementing a PGI (Protected Geographical Indication) approach according to the types of *shubat*; and (iv) the establishment of standards with international recognition. 

## Figures and Tables

**Table 1:** Mean physico-chemical parameters of Kazakhstani *shubat* from different regions, seasons, and camel breeds (from Konuspayeva [7]).

#	Parameter	n *	Mean and SD	Max Value	Min Value
1	pH	22	4.08 ± 0.28	4.72	3.55
2	Dornic (0D)	30	139 ± 29	189	86
3	Thurner (0T)	18	165 ± 31	215	106
4	Proteins (%)	29	3.34 ± 0.84	5.63	1.19
5	Vitamin C (mg L^−1^)	24	156 ± 110	417	28
6	Ca (g L^−1^)	30	1.35 ± 0.22	1.88	1.03
7	P (g L^−1^)	30	0.99 ± 0.32	1.80	0.11
8	Fe (g L^−1^)	30	3.04 ± 1.95	9.10	0.80

* n—number of samples.

## Data Availability

No new data were created or analyzed in this study. Data sharing is not applicable to this article.
